# Long-Term Response to a Bioactive Biphasic Biomaterial in the Femoral Neck of Osteoporotic Rats

**DOI:** 10.1089/ten.tea.2020.0018

**Published:** 2020-10-19

**Authors:** Deepak Bushan Raina, Aurimas Širka, Irfan Qayoom, Arun Kumar Teotia, Yang Liu, Sarunas Tarasevicius, Kathleen Elizabeth Tanner, Hanna Isaksson, Ashok Kumar, Magnus Tägil, Lars Lidgren

**Affiliations:** ^1^ Department of Clinical Sciences Lund, Orthopedics, Faculty of Medicine, Lund University, Lund, Sweden.; ^2^ Department of Orthopedics and Traumatology, Lithuanian University of Health Sciences, Kaunas, Lithuania.; ^3^ Department of Biological Sciences and Bioengineering, Indian Institute of Technology Kanpur, Kanpur, India.; ^4^ Queen Mary University of London, School of Engineering and Materials Science and Institute of Bioengineering, London, United Kingdom.; ^5^ Department of Biomedical Engineering, Lund University, Lund, Sweden.

**Keywords:** femoral neck canal, osteoporosis, zoledronic acid, regenerative medicine, local delivery

## Abstract

Osteoporosis often leads to fragility fractures of the hip, resulting in impaired quality of life and increased mortality. Augmenting the proximal femur could be an attractive option for prevention of fracture or fixation device failure. We describe a tissue engineering based strategy to enhance long-term bone formation in the femoral neck of osteoporotic rats by locally delivering bioactive molecules; recombinant human bone morphogenic protein-2 (rhBMP-2), and zoledronic acid (ZA) by using a calcium sulfate/hydroxyapatite (CaS/HA) biomaterial. A defect was created by reaming the femoral neck canal of osteoporotic (OVX) rats and they were treated as follows: G1. Empty, G2. CaS/HA, G3. CaS/HA+Systemic ZA, G4. CaS/HA+Local ZA, and G5. CaS/HA+Local ZA+rhBMP-2. Bone formation was evaluated 6 months after treatment. Further, radioactively labeled ^14^C-ZA was used to study the bioavailability of ZA at the defect location, which was determined by using scintillation counting. Micro-CT indicated significantly higher bone volume in groups G4 and G5 compared with the other treatment groups. This was confirmed qualitatively by histological assessment. Addition of rhBMP-2 gave no additional benefit in this model. Local delivery of ZA performed better than systemic administration of ZA. Mechanical testing showed no differences between the groups, likely reflecting that the addition of bioactive molecules had limited effect on cortical bone or the choice of mechanical testing setup was not optimal. Scintillation counting revealed higher amounts of ^14^C-ZA present in the treated leg of G4 compared with its contralateral control and compared with G3, indicating that local ZA delivery can be used to achieve high local concentrations without causing a systemic effect. This long-term study shows that local delivery of ZA using a CaS/HA carrier can regenerate cancellous bone in the femoral neck canal and has clear implications for enhancing implant integration and fixation in fragile bone.

**Impact statement:**

This long-term study shows a promising method to enhance bone formation in the femoral neck canal of osteoporotic rats. The approach involves a ceramic carrier that facilitates bone regeneration via sustained delivery of bioactive molecules locally in the femoral neck. Further, the carrier acts as a local depot for bioactive molecule delivery for as long as 6 months with overall positive effect on bone regeneration. The results from this model can potentially be translated into the clinics for reinforcing the femoral neck or for enhancing implant anchorage in poor-quality osteoporotic bone, which presents a real clinical challenge in fragility fractures.

## Introduction

Fragility fractures of the hip are on the rise and with the current age quake the worldwide numbers are estimated to exceed 2.5 million by 2025 and double by 2050.^1,2^ Apart from patient morbidity and societal impact, the mortality rate 1 year after hip fracture is a staggering 30%.^3^ More than half of the patients never return to their prefracture level mobility, a disastrous outcome in old age.^4^

Prevention of osteoporotic fractures has hitherto focused on pharmacological intervention with bisphosphonates or more recently by using biological drugs targeting osteoclasts with anti-RANKL treatment.^5^ Systemic use of bisphosphonates such as zoledronic acid (ZA) has shown promising results,^6^ whereas side-effects, including overall reduced remodeling of the skeleton, have raised concerns and lead to a decline in patient adherence to treatment.^7,8^ Bone remodeling is an important physiological process for the maintenance of bone strength, as long-term bisphosphonate therapy has been associated with atypical shaft fractures in long bones.^9^ Another approach to prevent osteoporotic fractures of the hip or to reduce fixation failure in osteoporotic bone could be to reinforce the femoral neck by using inherently strong biocompatible materials that remodel into living bone with passage of time. We have recently developed an animal model to study this phenomenon in the femoral neck canal of osteoporotic rats^10^ and augmentation led to new bone formation, which could possibly enhance the mechanical strength of the bone or improve bone-implant anchorage in osteoporotic patients.

Since bisphosphonates have shown to be effective, recent preclinical research has focused on delivering ZA systemically or locally at the desired site, primarily aimed at increasing implant anchorage with the surrounding bone.^11–13^ Studies have also indicated that local usage of low-dose ZA in cancellous bone, such as an experimentally created metaphyseal void, surprisingly leads to improved bone formation in rodent models.^14^ The notion of bisphosphonates such as ZA being only anti-catabolic needs to be redefined since they also impart a dose-dependent pseudo-anabolic effect on cancellous bone. Several preclinical trials with carriers containing ZA providing controlled local delivery have shown regeneration of cancellous bone without the addition of bone morphogenic proteins (BMPs).^13–15^

Thus, this study aimed at first evaluating the long-term (6 months) effect of local ZA delivery via a calcium sulfate/hydroxyapatite (CaS/HA) biomaterial on bone regeneration in a femoral neck defect model in osteoporotic rats. Second, we aimed at comparing local delivery of ZA with systemic ZA treatment and at evaluating the bioavailability of radioactively labeled ^14^C-ZA at the defect site during the entire duration of the experiment and its effect on bone formation. We hypothesized that the ZA-HA combination in the biphasic biomaterial will provide a sustained release of ZA, resulting in a positive effect on cancellous bone formation and that a long-term follow-up could increase the mechanical strength of the femoral neck canal in osteoporotic rats.

## Methods

### Study plan, experimental groups, and drug doses

**FIG. 1.**
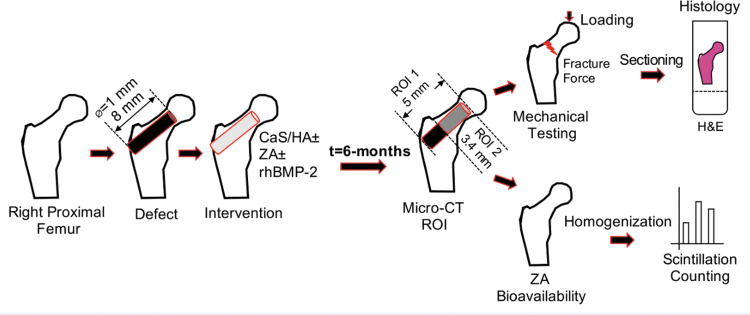
Schematic of the experimental study and the evaluation methods used for determination of bone formation, mechanical strength, and bioavailability of ZA. ZA, zoledronic acid. Color images are available online.

A defect was created in the femoral neck canal of ovariectomized (OVX) rats. The defect was either left empty or filled with a CaS/HA biomaterial with or without bioactive molecules (Fig. 1 and Table 1). After a period of 6 months, the animals were sacrificed and assessment of bone formation was performed by using micro-computed tomography (micro-CT), mechanical testing, and histology. Biodistribution of ZA was performed by using ^14^C-ZA either mixed in the CaS/HA biomaterial directly at the time of casting or administered systemically.

**Table 1.** Experimental Groups, Treatment, Bioactive Molecule Doses, and Sample Size for Each Evaluation Method in the Treated Leg

Group	Treatment (n)	Bioactive molecule dose/animal	Micro-CT (n)	Mechanical testing (*n*)	Histology (n)	Scintillation counting (n)
G1	Empty (8)	—	8	8	3	—
G2	CaS/HA (8)	—	6	6	3	—
G3	CaS/HA+Systemic ZA (12)	0.1 mg/kg ZA S.C 2-Weeks post-op	11	8	3	3
G4	CaS/HA+Local ZA (11)	10 μg ZA	11	8	3	3
G5	CaS/HA+Local ZA+rhBMP-2 (9)	10 μg ZA and 5 μg rhBMP-2	8	8	3	—

CaS/HA, calcium sulfate/hydroxyapatite biomaterial; n, the sample size; rhBMP-2, recombinant human bone morphogenic protein-2; S.C, sub-cutaneous; ZA, zoledronic acid.

### Preparation of CaS/HA biomaterial

CaS/HA biomaterial (G2 and G3) was prepared by mixing 1 g of the powder component (consisting of 60% CaS and 40% HA) with 112.5 μL saline and 317.5 μL of contrast agent (Iohexol^®^). After mixing the two components for 30 s, the biomaterial slurry was transferred to a graduated 1-mL syringe and portions of ∼55 μL were collected in a sterile dish. This process was repeated until all required specimens could be produced. After a period of 15 min, the biomaterial solidified due to the setting of the CaS phase. At this point, the biomaterial was divided further into small pieces by using a sharp scalpel and all of the material was impacted into the femoral neck canal defect (55 μL/animal) by using a steel rod. In G4, 1 g of CaS/HA powder was mixed with 112.5 μL ZA (90 μg, concentration: 4 mg/5 mL) and 317.5 μL contrast agent. A total of eight portions of 55 μL were obtained in this fashion whereas material slurry for approximately one portion was lost during the mixing and extrusion procedure. Each portion contained ∼10 μg ZA, and the procedure was repeated until the desired CaS/HA+ZA specimens were obtained. Similarly, G5 samples were achieved by mixing 1 g of CaS/HA powder with 112.5 μL ZA (90 μg, concentration: 4 mg/5 mL), 317.5 μL contrast agent, and 45 μg recombinant human bone morphogenic protein-2 (rhBMP-2). Each portion consisted of 55 μL CaS/HA slurry containing 10 μg ZA and 5 μg rhBMP-2. Animals in G4 and G5 received a total of 55 μL CaS/HA slurry and respective bioactive molecules/animal.

### Animal model and surgery

Bilateral ovariectomy was performed on female Sprague-Dawley rats at 12 weeks of age (Central Drug Research Institute, Lucknow, India). The interval between OVX and surgery was 16 weeks based on existing literature,^16^ as changes in cancellous bone architecture are prominent after this period. The animals were 28 weeks old at the time of operation and weighed 420 ± 20 g. Isoflurane anesthesia (2–4%) mixed with O_2_ was used during the entire duration of the surgery. Preoperative antibiotic prophylaxis in the form of ceftriaxone (40 mg/kg) was delivered intramuscularly 15 min before surgery, and diclofenac sodium (5 mg/kg) was used for postoperative pain relief.

The detailed surgical procedure is described in our earlier study.^10^ Briefly, after exposing the posterolateral aspect of the right femur, the leg was internally rotated to gain access to the inter-trochanteric crest. A 1-mm Ø drill bit was used to create a cylindrical cancellous bone defect running through the entire length of the femoral neck canal until the base of the sub-capital zone. The defect depth was ∼8 mm. At this point, the defect was treated as per the groups mentioned in Table 1. In the empty group, the defect was not treated with the CaS/HA material or bioactive molecules. In G3, nine animals were given a subcutaneous injection of ZA (0.1 mg/kg), 2 weeks post material implantation. All animals were sacrificed after an observation period of 6 months by using CO_2_ asphyxiation. Samples were kept frozen at −20°C in sterile saline-soaked gauze until further analysis.

### Micro-CT

Details of sample size for micro-CT are provided in Table 1. Harvested femora were thawed before imaging, individually placed in test tubes, and secured with the help of saline-soaked gauze. Samples were mounted on the stage of the instrument (Skyscan 1172; Bruker, Belgium) by using wax and scanned with the following settings: X-ray energy = 50 kV, current: 200 μA, exposure time = 800 ms, voxel size = 10 μm isotopic. X-ray projections were post-reconstructed by using NRecon software package (Bruker). All images were re-aligned by using Data Viewer (Bruker) along the long axis of the cylindrical defect. Region of interests (ROIs) were defined similar as in our earlier study.^10^ Briefly, two ROIs were chosen for analysis of bone regeneration, both with a diameter of 1 mm: (1) ROI1 began from the base of the sub-capital zone and ended near the point of entry (5 mm long) and (2) ROI2 started at the same position but included only the femoral neck canal (3.4 mm long) (Fig. 1). The image analysis was performed by using CTAn (Bruker). All images were thresholded between grayscale 75 and 255 for quantification of bone regeneration within the defect ROIs, and bone volume (BV)/tissue volume (TV) was used as the primary outcome variable from the micro-CT analysis. Antero-posterior X-ray projections from the micro-CT imaging were also used to obtain plain radiographs. After completion of micro-CT analysis, the samples were refrozen at −20°C.

As a surrogate for cortical thickness analysis, micro-CT images were also used to quantify the moment of inertia in the femoral neck. This was performed by identifying the starting point of analysis, which was 0.5 mm distal to the start of the femoral neck canal. Overall, 50 micro-CT slices were saved for each specimen from the starting point and an in-house MATLAB script was used to compute the moment of inertia on the saved slices.

### Mechanical testing

Femurs were thawed to room temperature 1 day before mechanical testing by placing them at 4°C. Thawed femurs were cut in the mid-diaphysis, and the distal femur was discarded. Samples were mounted in a Jacobs chuck that was connected to the stationary part of the mechanical testing equipment (Intron^®^ 8511 bi-axial load frame, 250 N load cell) in a configuration that enabled maintaining the long axis of the femur at 9° in adduction with respect to the vertical plane. The samples were compressed with a flat-end indenter at a displacement rate of 0.1 mm/s fracture (Fig. 1). The load-displacement data were used to determine the peak force to fracture. The location of fracture for each specimen was also recorded and was classified as either lateral or sub-capital.

### Histology

After mechanical testing, the broken femurs were formalin fixed overnight and decalcified by using an acid-based decalcification medium (Decalcification Solution-Lite; Sigma-Aldrich) for 48 h. Samples were embedded in paraffin by using routine histological procedures, including dehydration in increasing ethanol concentrations and clearance in xylene. Samples were infiltrated with molten paraffin overnight at 60°C with a change of paraffin the next morning and finally embedded in paraffin. Blocks were allowed to solidify for 1 day, after which 5–7 μm sections were obtained on a semi-automatic microtome (Thermo Scientific) and stained with hematoxylin and eosin (H&E) for microscopically evaluating the type of regenerated tissue in the femoral neck canal.

### Bioavailability of ZA

^14^C-labeled ZA (^14^C-ZA) (concentration: 1 mg/mL, specific radioactivity: 7.2 MBq/mL) was used in a total of six animals (*n* = 3 from G3 and *n* = 3 from G4). Biomaterial preparation and implantation was performed as described in the Preparation of CaS/HA Biomaterial section and the Animal Model and Surgery section. In G3, 2 weeks post-CaS/HA biomaterial implantation, animals received a single subcutaneous dose of ^14^C-ZA (0.1 mg/kg). In G4, instead of adding nonlabeled ZA to the CaS/HA biomaterial, ^14^C-ZA was directly mixed with the material and implanted in the femoral neck defect. Total volume of the implanted material and the dose of delivered ZA remained the same as described earlier. At the time of sacrifice and post**–**micro-CT imaging, the femurs (both treated and contralateral) were cut through the diaphyseal bone just below the starting point of the defect. The bones were cleaned of the surrounding soft tissue, and the weight of each bone specimen was recorded. All specimens were immersed in 5 M HCl for rapid decalcification, homogenized by using an ultrasound homogenizer, and mixed with scintillation cocktail for radioactivity detection by using scintillation counting (Wallac 1414; Perkin Elmer). Counts were measured during 120 s, and the scintillation cocktail alone was used for blank correction. Measured radioactivity was normalized to the weight of the bones and expressed as disintegrations per minute (DPM)/mg of tissue.

### Statistics

First, the data were tested for normality by using the Shapiro–Wilk normality test. Parametrically distributed datasets emanating from multiple groups were tested by using one-way ANOVA with Tukey's *post hoc* method. Nonparametric data were tested by using Kruskal–Wallis test with Dunn's correction; *p* < 0.05 was considered statistically significant. Paired samples were tested by using either a paired-sample *t*-test or Wilcoxon signed-rank test. Data are represented as mean ± standard deviation.

### Animal ethics statement

Animal experiments were approved by the Institute Animal Ethics Committee (IAEC) guidelines (Protocol number: IITK/IAEC/1062) approved by the Indian Institute of Technology Kanpur, India. ARRIVE guidelines have been followed for description of the animal study.

## Results

### Radiography and micro-CT

**FIG. 2.**
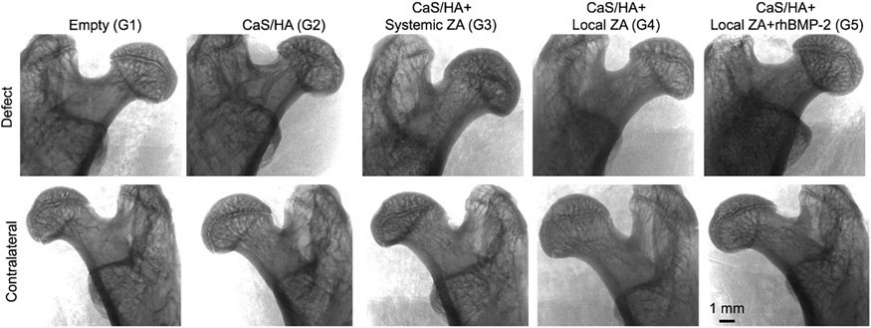
Plain X-ray radiographs of the treated (*top*) and contralateral (*bottom*) legs. Notice the increased radiodensity in the femoral neck region in CaS/HA+Local ZA and CaS/HA+Local ZA+rhBMP-2 groups in the treated legs. Scale bar represents 1 mm. Samples were randomly chosen for representation. CaS/HA, calcium sulfate/hydroxyapatite; rhBMP-2, recombinant human bone morphogenic protein-2; ZA, zoledronic acid.

X-ray images of the femoral neck area indicated similar radiopacity in the contralateral legs. Treated legs in G4 and G5 indicated increased radiodensity in the femoral neck canal compared with the rest of the groups (Fig. 2).

**FIG. 3.**
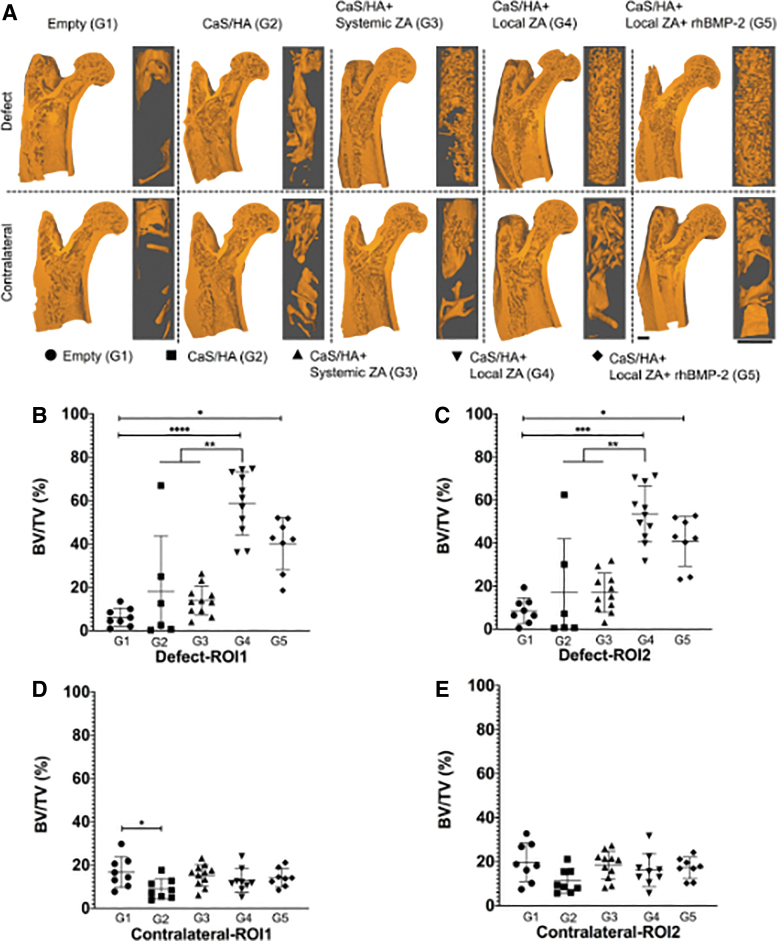
Micro-CT analysis. **(A)** Representative 3D reconstructions of the entire proximal femur (*left*) and the tissue segmented for BV calculations based on the dimensions of ROI1 (*inset*). **(B, C)** BV/TV quantifications from the micro-CT analysis in both ROI1 and ROI2 of the treated legs. **(D, E)** BV/TV quantifications from the micro-CT analysis in both ROI1 and ROI2 of the contralateral legs. Scale bars in **A** indicate 1 mm, *p < 0.05, **p < 0.01, ***p < 0.001, and ****p < 0.0001. BV, bone volume; micro-CT, micro-computed tomography; ROI, region of interests; TV, tissue volume. Color images are available online.

Treated leg: A similar effect on BV/TV was observed in both ROIs. After 6 months of surgery, the BV/TV (%) in ROI1 was significantly higher in G4 compared with G1 (*p* < 0.0001), G2 (*p* < 0.01), and G3 (*p* < 0.01) (Fig. 3A, B). BV/TV was also higher in G5 compared with G1 (*p* < 0.01). In ROI2, G4 had significantly higher BV/TV compared with G1 (*p* < 0.001), G2 (*p* < 0.01), and G3 (*p* < 0.01). G5 also had significantly higher BV/TV compared with G1 (*p* < 0.05). No other differences in the BV/TV were seen in the other groups (Fig. 3C).

Contralateral leg: G1 had significantly higher BV/TV (%) compared with G2 (*p* < 0.05) in ROI1 (Fig. 3D). BV/TV was similar in all other groups in both ROI1 (Fig. 3D) and ROI2 (Fig. 3E).

**FIG. 4.**
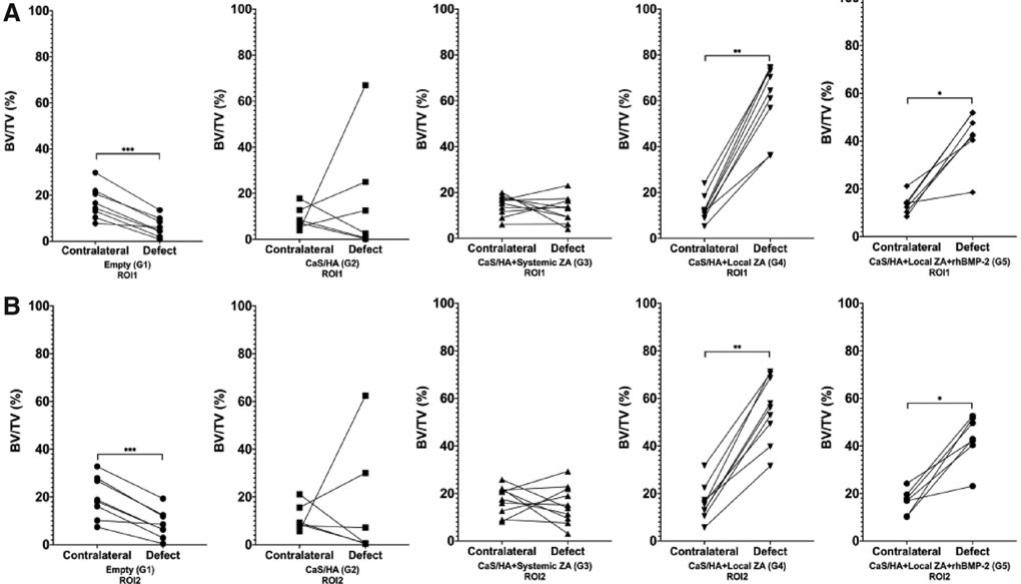
Micro-CT analysis of the treated and contralateral femurs. **(A)** Shows data from ROI1, **(B)** shows data from ROI2. Data points represent paired samples. *p < 0.05, **p < 0.01, and ***p < 0.001.

Comparison of treated versus contralateral legs: In ROI1, BV/TV in G1 was significantly higher in the contralateral leg compared with the treated leg (*p* < 0.001) (Fig. 4A). In both G4 and G5, BV/TV was higher in the treated legs compared with contralateral legs (*p* < 0.01 and *p* < 0.05, respectively) (Fig. 4A). No differences in treated and contralateral legs could be observed in G2 and G3. Similar results were observed for ROI2 (Fig. 4B).

### Mechanical testing and moment of inertia

**FIG. 5.**
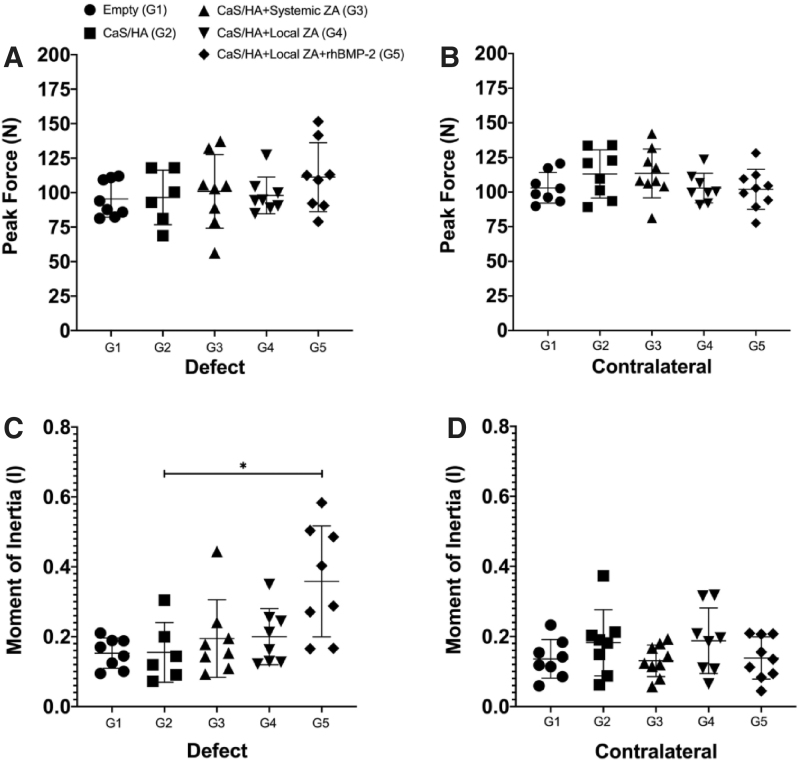
Mechanical testing and moment of inertia. **(A, B)** Peak force to fracture of the bones in the different treatment groups in the treated and the contralateral femurs, respectively. **(C, D)** Moment of inertia measured in the femoral neck in both treated and contralateral femurs, respectively.

Treated leg: The peak force to fracture of the treated legs in all groups was similar (Fig. 5A). Contralateral leg: No differences in the peak force could be observed in the contralateral leg (Fig. 5B). Comparison of treated versus contralateral legs: No differences in the peak force of the treated versus contralateral legs could be observed in any of the treatment groups (Supplementary Fig. S1). Moment of inertia was significantly higher in G5 compared with G2 (*p* < 0.05) in the treated leg (Fig. 5C). No other changes in moment of inertia were observed in any other treatment groups in both defect and contralateral legs (Fig. 5C, D).

Fracture location: Irrespective of the treatment group, the majority of the contralateral femurs fractured in the sub-capital region (Table 2). The creation of a defect in the femur and concomitant bone regeneration in some groups led to a change in the fracture location pattern. G1 femurs almost always fractured in the lateral location, whereas the other groups had a mixed pattern of lateral and sub-capital fractures.

**Table 2.** Fracture Location of the Femurs After Biomechanical Testing

Groups	Contralateral		Defect	
	Lateral (%)	Sub-capital (%)	Lateral (%)	Sub-capital (%)
G1	12.5	87.5	87.5	12.5
G2	12.5	87.5	50	50
G3	22	78	37.5	62.5
G4	25	75	50	50
G5	0	100	67	33

### Histology

**FIG. 6.**
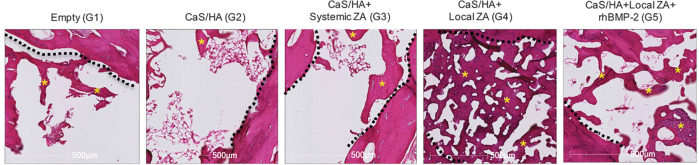
Representative H&E staining of the femurs after mechanical testing. Images have been taken from the femoral neck region. *Dashed black line* represents original cortical bone of the femoral neck. *Indicates bone tissue. H&E, hematoxylin and eosin. Color images are available online.

In G1, most of the defect area including the trochanteric region and the femoral neck canal were empty and sparsely covered with viable bone tissue (Fig. 6). Similar results were seen for G2 and G3. Both G4 and G5 demonstrated significant new bone formation in the femoral neck canal and trochanteric regions.

### Bioavailability of ^14^C-ZA

**FIG. 7.**
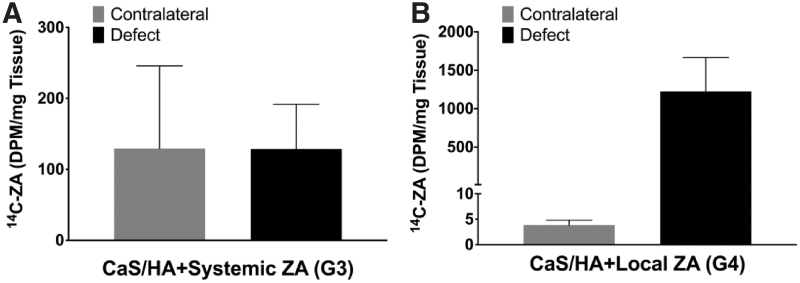
Bioavailability of ^14^C-ZA in the femur 6 months after surgery analyzed by using scintillation counting. **(A)** Shows data from G3, where ^14^C-ZA was systemically administered to the animals 2 weeks post**–**CaS/HA biomaterial implantation. **(B)** Shows data from G4 where ^14^C-ZA was mixed in the CaS/HA biomaterial and locally delivered.

Scintillation counting revealed similar amounts of ^14^C-ZA in the treated and contralateral legs of the CaS/HA+Systemic ZA (G3) group (Fig. 7A). Despite removing all cancellous bone from the defect leg, the presence of ^14^C-ZA after a 6-month period could be detected because of the presence of HA from the CaS/HA biomaterial within the defect.

In the case of locally delivered ZA from the CaS/HA biomaterial in the defect, higher counts of ^14^C-ZA were observed in the treated leg (Fig. 7B). The contralateral legs in this group demonstrated counts similar to the background control, revealing the very localized effect of this delivery method with this carrier.

## Discussion

In this study, we show increased bone regeneration in the femoral neck canal of osteoporotic rats after prolonged treatment (6 months) with locally delivered ZA or a combination of ZA and rhBMP-2 using CaS/HA biomaterial as a carrier. The animal model was recently described and reported at short-term follow-up (2-months), where we found that the CaS/HA carrier alone had a limited effect on bone formation in the reamed femoral neck defect.^10^ The short-term study indicated that local delivery of ZA alone was sufficient to significantly enhance the cancellous bone regeneration in the femoral neck canal without having to use rhBMP-2. Moreover, despite significant increase in bone regeneration, the earlier study could not verify that local bioactive molecule delivery had a positive effect on the mechanical strength of the femoral neck canal. It was, thus, imperative to evaluate the long-term effect of local ZA delivery on both bone regeneration and mechanical properties in this model. It is clear from the results from the two studies that rhBMP-2 does not necessarily add any beneficial effects in this model when compared with locally delivered ZA. Further, it is also shown that systemically administered ZA at a dose three to four times higher (0.1 mg/kg) than locally delivered ZA (10 μg) did not enhance bone regeneration in this model where a significant proportion of the cancellous bone in the femoral canal was removed at the time of the operation. This is contrary to the clear positive effect we found, using systemic ZA, on cancellous bone around a fenestrated implant containing the ceramic carrier in an implant integration study.^13^ Mechanical testing did not show any differences in any of the treatment groups. This could be because the augmentation method described in this study only improved cancellous bone regeneration and had a minor effect on the cortical bone, which accounts for about 80% of the mechanical strength in the cervical neck of people.^17^ Interestingly, the weakest point or the fracture location shift was observed in the defect legs of treatment groups that showed significant bone regeneration. Compared with the contralateral femurs that always fractured in the sub-capital region, the fracture location in the treatment legs in ZA (G4) and ZA+rhBMP-2 (G5) treated animals showed a mix of both lateral and sub-capital fractures. Whether this shift in fracture pattern can have a clinically relevant impact is speculative and more studies are required. It is also interesting to discuss that despite an increase in the moment of inertia in G5 compared with G2, no changes in peak fracture force were seen. This finding indicates that the mechanical testing set-up used in the study might not have been optimal to reflect the biomechanical changes caused by this augmentation method. Another possible factor that could have led to unchanged mechanical properties may be the duration after ovariectomy, which would be less likely to affect the cortical thickness.^16^ However, to make a comparison between this long-term study and the earlier study, it was important to keep the experimental conditions as similar as possible.

An important aspect of this study was to evaluate the bioavailability of ZA over a long period, primarily since long-term effects of local ZA delivery are not documented in the literature. Several preclinical studies have described local usage of ZA in studies involving bone defect regeneration,^14^ evaluation of carrier properties of scaffolds^18,19^ as well as in implant fixation studies,^11–13^ but little is known about the eventual long-term fate of the drug. Clinical pharmacokinetics of systemically administered ZA is well established in the literature^20^ but before clinical translation of locally administered ZA using a biomaterial carrier is possible, it is important to study the biodistribution of the drug and the resultant biological consequences. Systemically administered ZA preferentially binds to the skeleton and bone mineral and the rest is excreted within the first week of administration.^20,21^ Short-term studies, including delivery of ZA from a collagen-HA biomaterial,^18^ CaS/HA biomaterial,^13^ and gelatin-CaS/HA biomaterial,^19^ have been reported *in vivo* over a period of 24 h to 28 days by using radioactively marked ^14^C-ZA. All studies indicate a strong interaction between ZA and synthetic particulate HA. This strong interaction of ZA with HA has also been verified recently wherein it was demonstrated that even systemically circulating ZA can be recruited by locally implanted synthetic HA particles at both ectopic and entopic locations and this seeking phenomenon even exerts a biological effect.^22^ Long-term binding and the effect of ZA released from a biomaterial scaffold has not been performed earlier. The results from this study indicate that locally delivered ^14^C-ZA from a CaS/HA biomaterial remains in the biomaterial even after an implantation period of 6 months, consolidating a strong interaction of synthetic HA and ZA. Based on the bioavailability of ZA at the defect site for a long duration, it can be ascertained that increased bone formation in the animals treated with locally delivered ZA is, indeed, due to a pseudo-anabolic effect imparted by ZA on cancellous bone. Despite injecting a higher dose of ZA systemically, radioactive counts were lower in the systemic ZA group compared with the locally delivered group. This demonstrates the efficacy of local ZA delivery at the lower doses chosen in this study. It would be worth investigating the consequences of different ZA doses delivered locally by using biomaterial carriers before clinical translation. Earlier studies investigated whether local ZA delivery could circumvent side-effects of long-term systemically administered ZA, mainly reduce bone remodeling, and reduce overall ZA doses. Comparison of local and systemic ZA delivery in this model, indeed, indicates that local ZA delivery has a better outcome compared with systemic ZA delivery but needs to be carefully interpreted, as they might not translate to defects at other anatomical locations. Further, experiments involving radioactive ZA clearly show that when ZA is delivered locally, the drug does not leak out into the systemic circulation since the contralateral femur shows counts corresponding to background radioactivity. This result is particularly important in minimizing the risk for atypical cortical fractures associated with long-term systemically administered bisphosphonates that are commonly being reported in the recent literature.^9^

Mechanical reinforcement of the femur can be performed either with inherently strong materials, such as bone cement, or with materials that can initiate new bone formation. In a study by Cheng *et al.*, bone cement poly methyl methacrylate (PMMA) was injected in elderly patients with osteoporosis undergoing fixation of trochanteric fractures.^23^ Excellent to good outcome was reported in 75% of the cases whereas the remaining patients had complications. Building up on existing literature and the key findings from Cheng *et al.*, Sutter *et al.* restricted the volume of PMMA to 15 mL/bone to minimize heat induced necrosis, prevent emboli formation, and make revision surgery easier.^24^ They used osteoporotic human cadaveric bones and injected bone cement either in the femoral neck region or in the trochanteric region and used contralateral femurs as controls. They did not observe any differences in the mechanical properties of the femurs after this augmentation procedure.^24^ Other cadaveric studies performed with larger volumes of PMMA have shown an increase in mechanical characteristics of bone but potential risks, especially with high exothermic temperatures, do not outweigh the benefits.^25^ It is also important to mention that although PMMA is an inert, biocompatible, and mechanically strong material, it also is a “dead” material that does not remodel into living bone, or stimulate new bone formation.

One of the goals of this study was to develop a method to reinforce the femur with a bioactive biomaterial during the initial phase, which would eventually remodel into living bone and enhance the biomechanical properties of the proximal femur. This could potentially be used in enhancing integration of orthopedic hardware such as implants, nails, and screws. During late-stage osteoporosis, the micro-architecture and mechanical properties of bone around the fixation device are compromised; therefore, screws or nails fail to provide initial stability, leading to failure and re-operation in up to 10–25% of patients.^26,27^ Based on the results from this study, it is likely that a CaS/HA biomaterial locally releasing ZA around an implant could enhance peri-implant cancellous bone formation. We recently described this concept in an implant integration model in healthy male rats by using a fenestrated implant filled with a CaS/HA biomaterial releasing ZA,^13^ but the concept remains untested in osteoporotic animals, especially over a longer period. However, it might be more feasible to use a regular cannulated implant reinforced with CaS/HA biomaterial delivering local ZA in the surrounding bone, from a perspective of both enhanced initial mechanical stability and cost to the health care system. It is also important to emphasize that sufficient cancellous bone regeneration can be achieved by using controlled local delivery of ZA alone, also shown earlier, and thus use of rhBMP-2 can completely be avoided in such circumstances.

Although augmentation of the femur using bone cement was described nearly three decades ago, scientists recently have started exploring the possibility of using bioactive materials such as hydroxyapatite, calcium phosphates, and calcium sulfates as alternatives for enhancing the mechanical strength of bones.^28^ Most of these studies have, however, investigated the tibial plateau fractures. A recent experimental/computational modeling study by Kok *et al.* assessed the feasibility of injecting a CaS/HA biomaterial into the trochanter and femoral neck canal of patients undergoing total hip arthroplasty and concluded that it was feasible to inject up to 10 mL of the biomaterial.^29^ Further, using finite element modeling, the study also indicated that the location of injection and the volume of the injected material were important factors for enhancing the mechanical properties of the femur. However, the simulations could not account for or predict the *in vivo* CaS/HA biomaterial degradation or the bone remodeling. In a clinical study by Stravinskas *et al.*, a gentamycin containing CaS/HA biomaterial was injected into the trochanter to enhance fracture fixation in patients undergoing treatment for trochanteric fractures or revision hip arthroplasty.^30^ At a one-year follow-up, minimal screw subsidence was noticed with radiological signs of new bone formation around the bone substitute without the addition of any bioactive molecules. The results from this study indicate that local ZA delivery from a CaS/HA biomaterial could further improve the bone regeneration potential and long-term fixation, for which further studies are warranted.

## Conclusions

In conclusion, long-term local delivery of ZA via a CaS/HA biomaterial promotes cancellous bone regeneration in the femoral neck canal of osteoporotic rats and no additional osteoinductive factors such as BMPs are required. Long-term exposure to a high local concentration of ZA did not have any negative impact on bone. Regenerating large volumes of cancellous bone in this femoral neck canal model did not enhance the mechanical strength of the femur, which could be attributed to the mechanical testing set-up or the inability of the treatment to significantly affect the strength of the cortical bone in the femoral neck. However, a shift in the weakest point in the femur could be observed as contralateral femurs broke sub-capitally whereas treated femurs demonstrated a mixture of lateral and sub-capital fractures. Biodistribution analysis using ^14^C-ZA indicated that locally delivered ZA using a CaS/HA biomaterial remains at the implantation site for at least up to 6 months and higher counts of ZA were found at the defect site compared with systemically administered ^14^C-ZA, which was administered at a dose three to four times higher than local ZA. Further, locally administered ZA did not cross over to the contralateral leg, showing a very localized delivery effect. Our results can potentially transfer to the clinical setting and aid in enhancing fracture fixation in osteoporotic patients for which further studies are warranted.

## Supplementary Material

Supplemental data
