# Supplementary material for: Long-Term Response to a Bioactive Biphasic Biomaterial in the Femoral Neck of Osteoporotic Rats
Source: Tissue Eng Part A. 2020 Oct 19;26(19-20):1042–51. doi: 10.1089/ten.tea.2020.0018 (PMC7580608; doi:10.1089/ten.tea.2020.0018)
Supplement: Supplemental data [file Supp_Fig1.pdf]

Supplementary Data

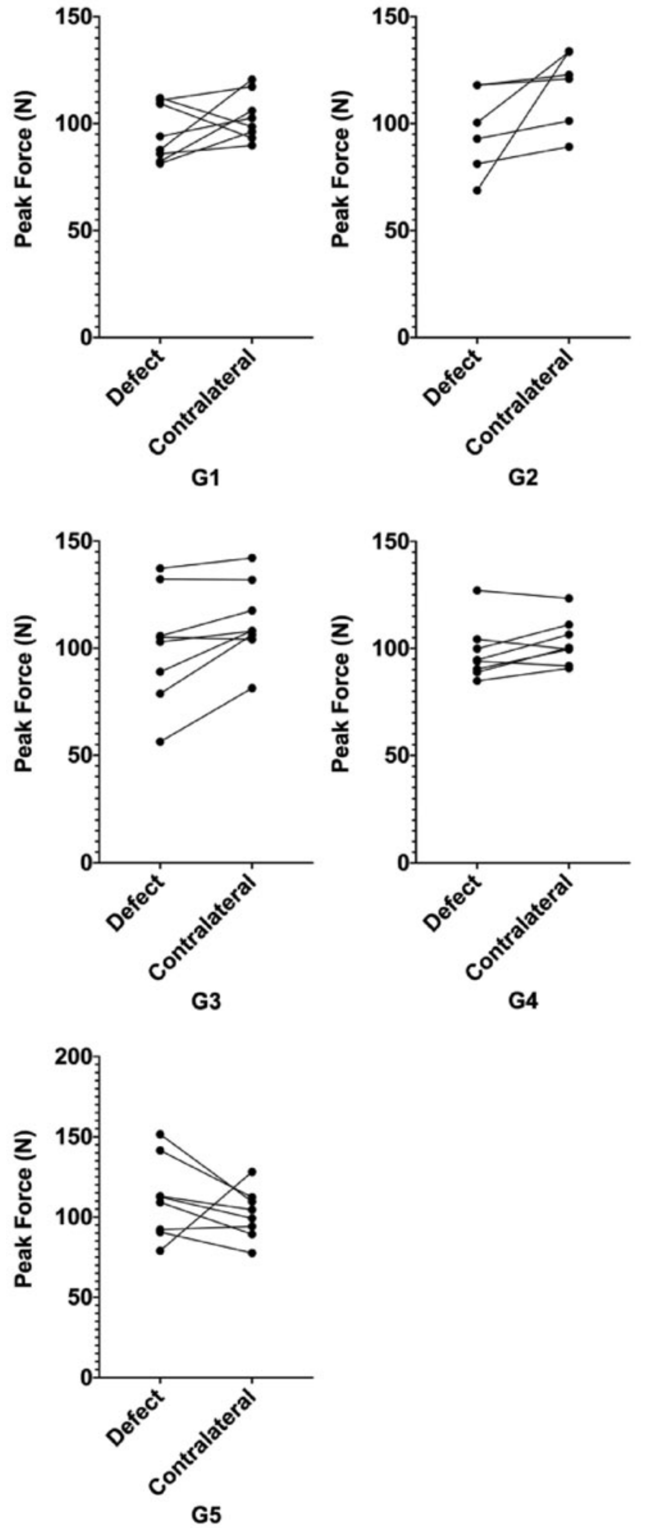

SUPPLEMENTARY FIG. S1. Comparison of peak force between the defect and contralateral legs in treatment groups G1–G5.
